# Prognostic relevance of specific TIL (CD4+, CD8+, and FOXP3 + T-cell infiltrates) in triple-negative breast cancer: short- and long-term outcomes

**DOI:** 10.1007/s12282-025-01819-y

**Published:** 2026-01-07

**Authors:** Olga Caramelo, Vânia Almeida, Ana Fidalgo, Augusta Cipriano, Teresa Almeida-Santos

**Affiliations:** 1 Department of Gynecology, Unidade Local de Saúde de Coimbra, Coimbra, Portugal; 2 Department of Pathology, Unidade Local de Saúde de Coimbra, Coimbra, Portugal; 3 Faculty of Medicine, University of Coimbra, Coimbra, Portugal; 4 Immunethep, Cantanhede, Portugal

**Keywords:** Breast cancer, Immune cells, TIL, T cells, CD4 + T cells, CD8 + T cells, T regs, FOXP3 + cells

## Abstract

**Background:**

Breast cancer is a heterogeneous malignant disease that remains as one of the most prevalent cancers globally. Triple negative breast cancer (TNBC) accounts for 15% of the total of breast cancers and presents high tumor immunogenicity and a tumor microenvironment that plays a critical role in disease progression and patient outcomes.

**Methods:**

This study evaluated a total of 30 tissue samples from female patients with TNBC, to characterize specific immune cells within the tumor tissue and investigate their relationship with short (pathological complete response, pCR), long (disease-free survival, DFS) and clinical outcomes. Tumor-infiltrating lymphocytes (TIL) were assessed by immunohistochemistry (IHC) on tissue microarrays (TMA), complemented by digital analysis for standardized quantification.

**Results:**

Our results highlight the influence of CD4⁺ T cells on short-term outcomes: high CD4⁺ T-cell levels were significantly associated with achieving pCR. High CD8⁺ T-cell levels were also significantly associated with axillary lymph node negativity.Regarding long-term outcomes, higher CD4⁺, CD8⁺ and FOXP3⁺ T-cell levels showed a non-significant tendency toward improved DFS.

**Conclusions:**

These findings suggest that high levels of CD4 + T cells and CD8 + T cells are positive predictors of immediate, long-term prognosis and clinical prognosis in patients with TNBC. This study enhances the understanding on the immunological interests in specific subtypes of TIL and identifies potential biomarkers that could drive advancements in precision medicine for breast cancer management.

**Supplementary Information:**

The online version contains supplementary material available at 10.1007/s12282-025-01819-y.

## Introduction

Approximately 15% of breast cancer cases are diagnosed as triple negative breast cancer (TNBC), which is recognized as a highly heterogenous disease [1]. Often TNBC exhibits a biologically aggressive behavior, resulting in a poor prognosis with a higher tendency to metastasize compared to other breast cancer subtypes [2, 3].

Until recently, chemotherapy was the only systemic treatment option for this subtype of breast cancer, posing significant challenges due to the exclusion of effective therapies such as hormone therapy and anti-HER2 agents [4].

The 5-year survival rate of TNBC is significantly lower when comparing to hormone receptor-positive breast cancer [5]. However, in the last decade substantial research has focused on improving the prognosis of TNBC, and newer treatment options, particularly immunotherapy, are being implemented in clinical practice [5].

The progression of breast cancer is complex and results from the interaction between tumor cells and components of the tumor microenvironment. Immune cells within this microenvironment play a critical role in the initiation and progression of breast cancer, as they can either promote or inhibit tumor development. Analyzing the immune response within tumor tissue can aid in predicting disease outcomes and planning treatment strategies. Recent research has emphasized that TIL significantly influence breast cancer management and the immune response overall [6]. TIL serve as important prognostic biomarkers and impact the efficacy of chemotherapy and immunotherapy, highlighting their significance in TNBC treatment [7]. TIL comprise a variety of immune cells that, based on phenotype, include CD8 + cytotoxic T cells, CD4 + helper T cells, CD4 + regulatory T cells or CD4 + follicular helper T cells. However, the specific role and clinical significance of TIL subpopulations are still uncertain [8]. In breast cancer, T cells constitute the vast majority of TIL accounting for up to 75% of the infiltrating immune population [9, 10].

While CD4 + T lymphocytes modulate host response upon MHC class II-restricted presentation of tumor-associated antigens, CD8 + cytotoxic T lymphocytes directly attack tumor cells through cell-mediated immune responses [11]. CD8 + cytotoxic T cells are key effectors of the adaptive immune system, capable of recognizing tumor antigens and playing a crucial role in tumor cell elimination. They are essential markers of anti-tumor immune responses, inducing apoptosis in target cells and producing pro-inflammatory molecules [12]. TNBC exhibits an enrichment of both CD8 + cytotoxic T cells and CD4 + regulatory T cells relative to other breast cancer subtypes [11], underscoring the importance of TIL, particularly CD4 + and CD8 + T cells, as predictive markers for neoadjuvant chemotherapy (NACT) response [10, 13, 14].

CD4 + T cells can be further subdivided upon activation into various functional subtypes of T helper (Th) cells, characterized by distinct patterns of interleukin production. They play a central role in anti-tumor activity by producing cytokines such as IFN-γ and TNF-α [15]. Regulatory CD4 + T lymphocytes (Tregs) are specialized CD4 + T cells characterized by the expression of forkhead box P3 (FOXP3) protein [16]. These cells are essential to main immune hemostasis and suppress pathogenic immune responses, modulating the immune response to prevent autoimmunity through their immunosuppressive function [17]. In the context of tumor, Tregs promote immune escape of tumor cells and are associated with advanced tumor stages and poor prognosis [16]. These cells suppress the activity of other immune cells through various mechanisms, including the release of soluble immunosuppressive cytokines like IL-10 and TGF-β, thereby facilitating tumor growth and metastasis [17].

The presence of TIL indicates a host immune response against tumor cells, with higher levels found in TNBC compared to other breast cancer types [18]. Moreover, TIL density in patients with TNBC is usually correlated with longer survival [19]. Furthermore, high density of TIL is correlated with significantly improved clinical outcomes and higher rates of pathologic complete response (pCR) following NACT, compared to lower TIL densities [20]. Approximately 10% to 20% of TNBC cases harbor *BRCA1* or *BRCA2* germline mutations [21]. These patients are candidates for treatment with poly (ADP-ribose) polymerase (PARP) inhibitors. The high immunogenicity and increased levels of TIL are key features of the immune landscape associated with TNBC. The remarkable response of TNBC to immunotherapy has transformed the standard of care in both early and metastatic settings [22].

This study aims to quantify and evaluate the impact of specific TIL, including CD4 + T cells, CD8 + cytotoxic T cells and Tregs in immediate and long-term prognostic outcomes such as pCR and disease-free survival (DFS) in patients with TNBC.

## Materials and methods

### Patients and sample selection

Formalin-fixed paraffin-embedded (FFPE) samples (*n* = 30) of triple negative breast cancer were obtained between January of 2015 and December of 2022 for this study. The specimens were obtained by biopsies of breast tumor.

All core biopsy prior to NACT and breast tumor excision specimens were fixed in 10% neutral buffered formalin and embedded in paraffin, and stored at the archive of the Anatomic Pathology Department. The ethical council of the institution reviewed and approved the protocol of this study.

The *pCR* of the surgically resected tumor was defined as the absence of all invasive cancer cells in both the breast and axillary lymph nodes, regardless of the presence of non-invasive cancer cells.

Reporting was revised to comply with the REMARK guidelines for tumor marker studies, including explicit details on follow-up time, number of events, and missing data.

### Staining and quantification of CD4, CD8 and FoxP3 T-cells

Tissue samples from breast cancer were fixed with 4% formaldehyde-buffered aqueous solution (VWR, Leuven, Belgium) following resection, in accordance with the procedures established at the Anatomic Pathology Department. Representative tumoral areas were selected by a pathologist based on 4 μm tissue sections previously stained with Hematoxylin-Eosin. A tissue microarray (TMA) was assembled using a 4 Ø mm biopsy dermal punch from the recipient paraffin block, which had been previously annotated to a receptor paraffin block holding 12 tissue cylinders from the representative samples. For each case, a representative tumor core was included in the TMA, selected on H&E slides by an experienced pathologist to contain viable invasive carcinoma with adjacent stroma. Within each TMA core, a single 500 μm × 500 μm region of interest (ROI) was manually placed in an area with a preserved tumor–stroma interface,avoiding necrosis, crush artefacts and tissue folds. In keeping with the recommendations of the International TIL Working Group for breast cancer,we focused our quantification on stromal TIL whenever possible, prioritizing ROIs with an abundant stromal compartment surrounding invasive tumor cell nests. In cores with more limited stroma, the ROI was centered on the most representative area of lymphocytic infiltrate at the invasive front.

Assembled TMA sections were stained with anti-CD3 Mouse Monoclonal (clone LN10, Leica, Breckland, United Kingdom)), anti-CD4 Rabbit Monoclonal (clone SP35, Roche, Basel, Switzerland), anti-CD8 Mouse Monoclonal (clone C8/144B, Dako, Næstved, Denmark), and anti-FoxP3 Mouse Monoclonal (clone 236 A/E7, Leica, Breckland, United Kingdom). Immunohistochemistry assays were performed on a BenchMark Ultra automated immunostainer (Ventana, Arizona, USA). Antigen retrieval was performed with an EDTA-Tris-based buffer pH8 (CC1, Ventana Medical Systems, Arizona, USA) at 95 °C, for 20 min. Detection of primary antibody was carried out using a brow color through a free-biotin Optiview DAB IHC Multimer Indirect Detection System Kit (Ventana Medical Systems, Arizona, USA). The slides were then counterstained with hematoxylin, dehydrated, and mounted with a synthetic mounting medium (Klinipath, Breda, Netherlands).

CD3 immunostaining was performed as a pan-T-cell marker and to confirm the overall distribution and technical adequacy of the lymphocytic infiltrate in each core. Because our primary objective was to assess the prognostic value of functionally distinct T-cell subsets (CD4⁺ helper, CD8⁺ cytotoxic and FOXP3⁺ regulatory T cells), and to avoid redundancy with these markers, CD3 was not included in the final quantitative statistical analysis.

Stained TMA slides were scanned using an Aperio Scanscope CS microscope (Leica, Wetzlar, Germany) at 20× objective (0.49 microns/pixel), and digital images were acquired for quantitative analysis. The quantification of positively stained cells was performed using the software package QuPath 0.4.3. (see https://qupath.github.io/). Cores were excluded if no analyzable tissue was present. Image type settings in QuPath were set to Brightfield (H-DAB). The total number of counted cells and positively stained cells (DAB) for each core were tabulated using the positive cell count function in QuPath (analyze - cell detection - positive cell detection) (Figure S1 – Supplementary data). For each core, a representative region of tissue measuring 500 μm × 500 μm was selected for analysis, avoiding areas of poor scan quality such as tissue folds. The percentage of positive cells was calculated using the formula: (positive cells / total cells) *100 (%). Quantification and grading were performed in a blinded fashion, separate from survival analysis.

Quantification of cell proliferation index Ki-67 was performed in tissues stained with Ventana Medical Systems, Inc. (30 − 9) Rabbit Monoclonal Primary Antibody using image analysis software.

### Statistical analysis

Statistical analyses were performed using GraphPad Prism software version 10.4 *and RStudio (running R version 4.5.1).*

Differences between the expression of TIL subtypes were evaluated using the Kruskal-Wallis test for unpaired data, with multiple comparisons corrected using Dunn’s test.

Comparisons of TIL percentages between cases with and without pCR were performed for each subtype using an unpaired t test with Welch’s correction. The unpaired t test with Welch’s correction was also applied to compare the percentage of CD8 + T cells between cases stratified by axillary lymph node status (positive vs. negative).

Correlations were calculated by Spearman’s correlation test.

Associations between TIL subtypes expression and clinical-pathological characteristics were evaluated by Fisher’s exact test. Percentages of TIL subtypes *were* dichotomized into low and high roups based on ROC curves using pCR as the outcome. Optimal thresholds were determined using the Youden index.

The influence of CD8 + T cell percentage on axillary metastasis was evaluated using univariate logistic regression analysis.

Kaplan-Meier estimation curves *were used to estimate* disease-free survival *and differences between groups were assessed using the log-rank test.*

All tests were two-sided and a p-value ≤ 0.05 was considered statistically significant.

## Result

### Clinical-pathological features and outcomes of patients

The characteristics of the cases used in this study are summarized in Table 1.

**Table 1:** Clinicopathological features of patients included in this study. Data are presented as median (interquartile range, IQR) for continuous variables and as number of cases and percentage (n (%)) for categorical variables. Features include age, clinical stage, histologic grade, Ki67 expression, histologic subtype, type of surgery, treatment modality, pathological complete response (pCR), presence of pathogenic BRCA1/2 variant, recurrence, and death

Clinicalpathological feature	Category	Median (IQR)	*n* (%)
Age, years		54, 47–66	30
Clinical stage	I		13 (43.3)
	II		12 (40.0)
	III		3 (10.0)
	IV		2 (6.67)
Histologic grade	2		7 (23.3)
	3		23 (76.7)
Ki67	High		24 (80.0)
	Low		6 (20.0)
Histologic subtype	Ductal		28 (93.3)
	Lobular		2 (6.67)
Surgery	Tumorectomy		18 (62.1)
	Mastectomy		11 (37.9)
Treatment	NACT		17 (56.6)
	Chemotherapy		11(36.6)
	Palliative Chemotherapy		2 (6.67)
pCR	Yes		10 (58.8)
	No		7 (41.2)
Pathogenic variant (BRCA 1/2)	Yes		5 (16.7)
	No		21 (70.0)
	unknown		4 (13.3)
Recurrence	Yes		4 (13.3)
	No		26 (86.7)
Death	Yes		4 (13.3)
	No		26 (86.7)

The median age of the 30 female patients identified as having TNBC was 54 years. Five patients (16.7%) were *BRCA*-mutated.

Regarding clinical stage, the vast majority of the cases were ductal invasive carcinoma (93.3%). Clinical stage I was represented in 43% of the cases, followed by stage II (40%). High histologic grade was observed in 76.7% of the cases. 62% of the patients underwent to tumorectomy.

Among the 17 patients who received neoadjuvant treatment, 10 (58.8%) achieved pCR, while 7 (41.2%) showed no or partial response. During follow-up, 4 patients (13.3%) experienced recurrence and died, while 26 patients (86.7%) remained disease-free.

### Analysis of the immune cell infiltrate composition

**Fig. 1:**
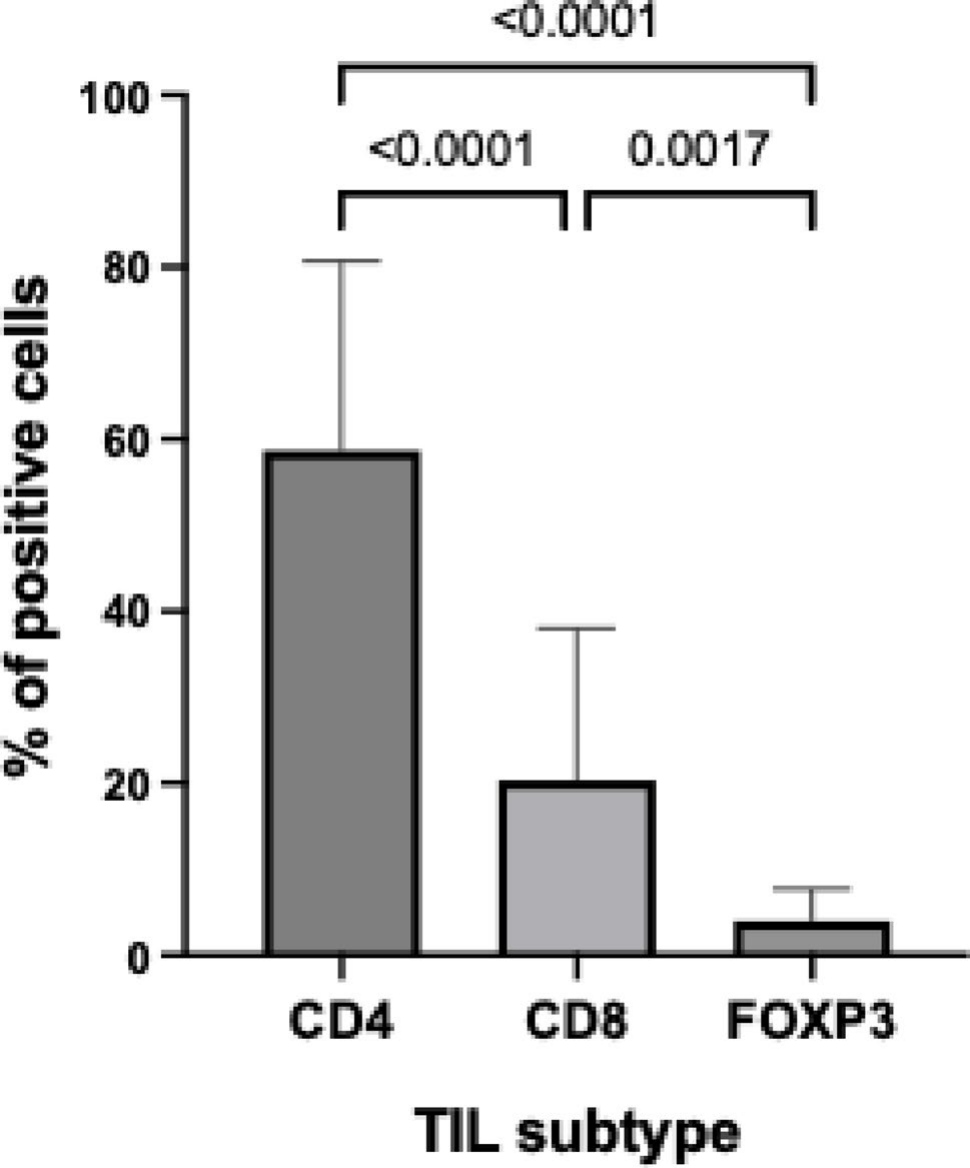
Percentage of tumor-infiltrating lymphocytes (TIL) positive for CD4+ (*n* = 30), CD8+ (*n* = 29) and FOXP3+ (*n* = 28) T cells. Bars represent the mean and the error bars the standard deviation. Differences between groups were evaluated using the Kruskal-Wallis test for unpaired data, with multiple comparisons corrected using Dunn’s test

Quantification of relative frequency of CD4 + T cells was achieved in all cases, while CD8 + T cells and FOXP3 + T cells were quantified in 29 and 28 cases, respectively. Regarding the distribution of TIL, samples showed a significant (*p* < 0.0001) enrichment of CD4 + T cells, with a mean frequency of 58.6% (± 22.1%), followed by CD8 + T cells at 20.3% (± 17.8%) and FOXP3 + T cells at 3.95% (± 3.80%) (Fig. 1). This highlights the marked predominance of CD4 + T cells in the tumor microenvironment compared to the other TIL subtypes.

### Differences in the frequency of TIL regarding the pCR

**Fig. 2:**
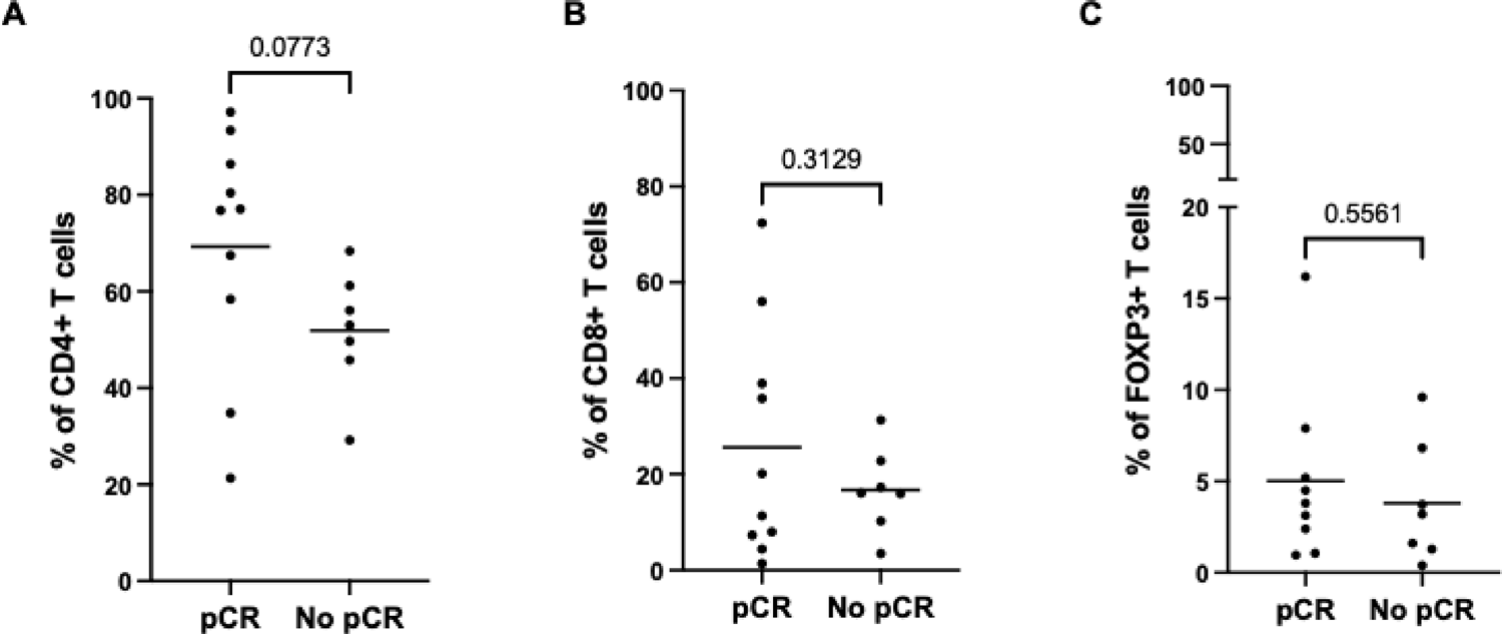
Percentage of tumor-infiltrating lymphocytes (TIL) positive for CD4+ (A), CD8+ (B) and FOXP3+ (C) T cells in cases with and without (no) pathological complete response (pCR). Differences between groups were evaluated using an unpaired t test with Welch’s correction. Sample sizes: CD4 + T cells — pCR (*n* = 10), no pCR (*n* = 7); CD8 + T cells — pCR (*n* = 10), no pCR (*n* = 7); FOXP3 + T cells — pCR (*n* = 9), no pCR (*n* = 7). Bars represent the mean of each group

Cases were divided in two groups according to pCR and differences in the expression of CD4+, CD8 + and FOXP3 + T cells were evaluated (Fig. 2). In the analysis of CD4 + T cells and CD8 + T cells frequencies, 10 and 7 patients were included in the group with pCR and without pCR, respectively. We observed a near-significant (*p* = 0.0773) increase in CD4 + T cells in cases with pCR comparing with cases without pCR, while no significant differences (*p* = 0.3129) were found in the frequency of CD8 + T cells between groups. Regarding FOXP3 + T cells, 9 cases from the pCR group and 7 from the group without pCR were included in the analysis but no significant (*p* = 0.5561) differences in the frequency of FOXP3 + T cells were observed.

### Correlation between TIL and immediate outcomes

A Spearman’s rank correlation analysis was performed to explore the relationship between the different TIL (CD4+, CD8 + and FOXP3 + T cells) and the cell proliferation marker KI67, in two groups based on whether pCR was achieved or not. 10 cases were included in the group with pCR (except in the analysis of FOXP3, which included 9 cases) and 7 cases were included in the group without pCR. The results are summarized in Table *S2 – Supplementary data*.

In the group of cases with pCR, we observed a significant strong positive correlation (ρ = 0.7455; *p* = 0.0174) between CD8 + T cells and CD4 + T cells and also between FOXP3 + and CD8 + T cells (ρ = 0.7833; *p* = 0.0172), suggesting that increases in these TIL subtypes are associated with each other.

In the group of cases without pCR, a significant strong negative correlation was found between CD4 + and CD8 + T cells (ρ = -0.8929; *p* = 0.0123), indicating that the relative frequency (%) of CD4 + T cells is consistently associated with lower percentages of CD8 + T cells. Also, a significant strong negative correlation was observed between percentage of CD8 + T cells and high KI67 (ρ =- 0.8469; *p* = 0.0246). Conversely, a positive strong correlation was identified between the percentage of CD4 + T cells and high KI67 (ρ = 0.8469; *p* = 0.0246).

### Association of TIL subtypes with clinical pathological characteristics

The frequency of TIL subtypes was dichotomized in low (< ) or high (≥) groups based on ROC-derived thresholds using pCR as the outcome: CD4 + T cells, 72.6%; CD8 + T cells, 9.12%; FOXP3 + T cells, 3.77%. For KI67, a threshold of 20% of expression was used to separate the groups in low (≤ 20%) or high (> 20%). The associations between dichotomized percentages of TIL subtypes and clinical-pathological characteristics - pCR, positive axillary lymph nodes, KI67 and degree - were analyzed, and results are presented in Table 2. We found a significant association between CD4 + T cells and pCR (p = 0.0345). Additionally, while not statistically significant (*p* = 0.0667), the percentage of CD*8* + T cells showed a marginally significant association *with negative axillary lymph nodes.*

**Table 2:**
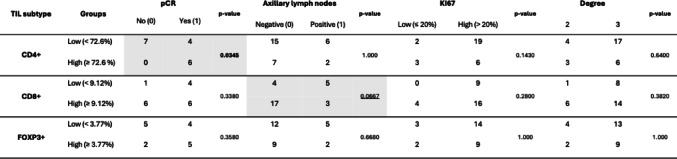
Association between low and high composition on CD4+, CD8 + and FOXP3 + T cells *(defined by receiver operating characteristic (ROC)-derived thresholds)* and clinical-pathological characteristics.

Values in each cell represent the number of individuals per category. Associations were evaluated *using* Fisher’s exact test. Statistically significant associations *(p-value ≤ 0.05)* are highlighted in bold, and marginally significant results (0.05 < *p* ≤ 0.10) are underlined.

### Exploring the association between CD8 + T cells and axillary metastasis.

**Fig. 3:**
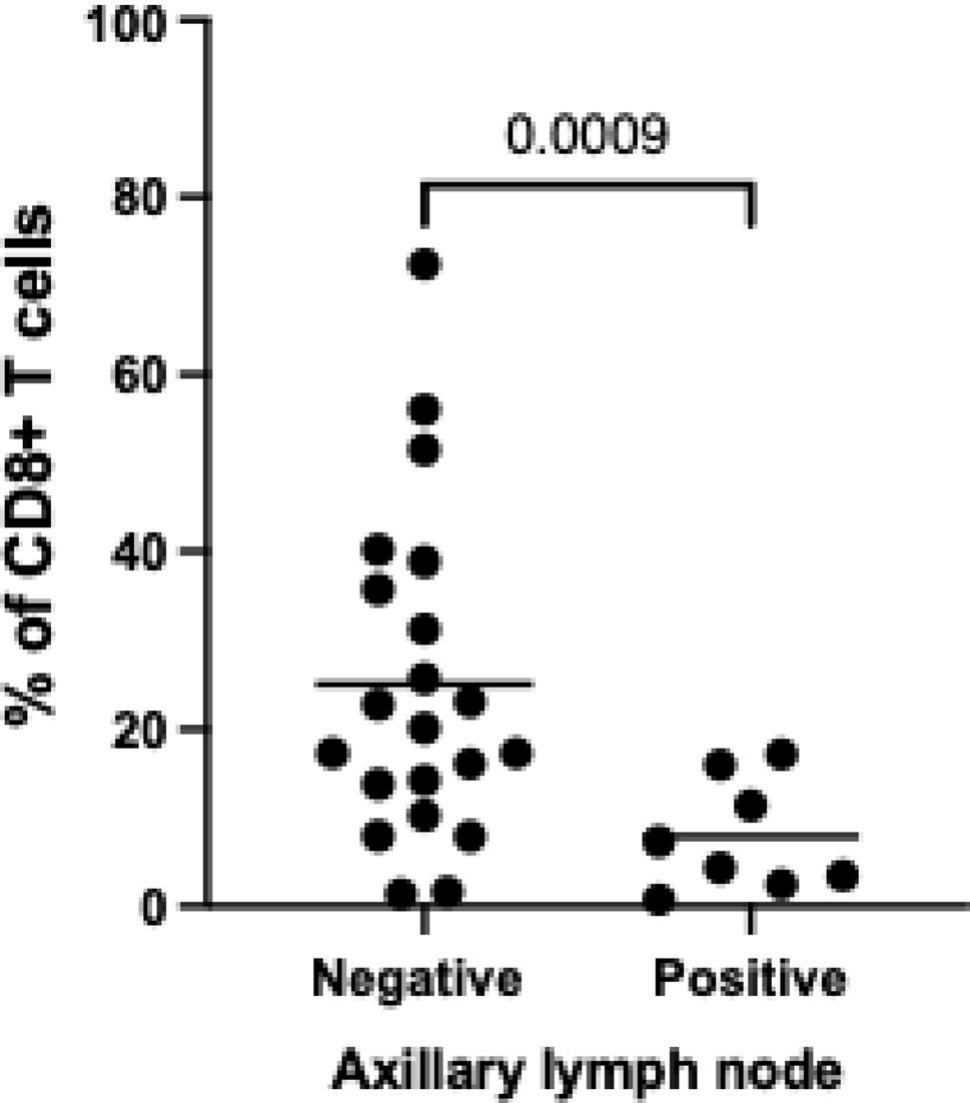
Association between CD8 + T cell percentage and axillary lymph node status. Percentage of CD8 + T cells in cases with negative (*n* = 8) and positive (*n =* 21) axillary lymph node status. Differences between cases were analyzed using the unpaired t test with Welch’s correction. Bars represent the mean of each group

*Given the marginal association observed between* CD8 + T cells and axillary metastasis, *we further explored this relationship using logistic regression by dichotomizing cases* on negative (n = *21*) or positive (n = *8*) lymph node status *and* compar*ing the groups* regarding the percentage of CD8 + T cells. As shown in Fig. 3, the percentage of CD8 + T cells *was* significantly higher in cases with negative axillary lymph nodes (*p* = 0.0009) compared to those with positive axillary lymph nodes, which reinforces the association between the percentage of CD8 + T cells and the axillary metastasis.

**Fig. 4:**
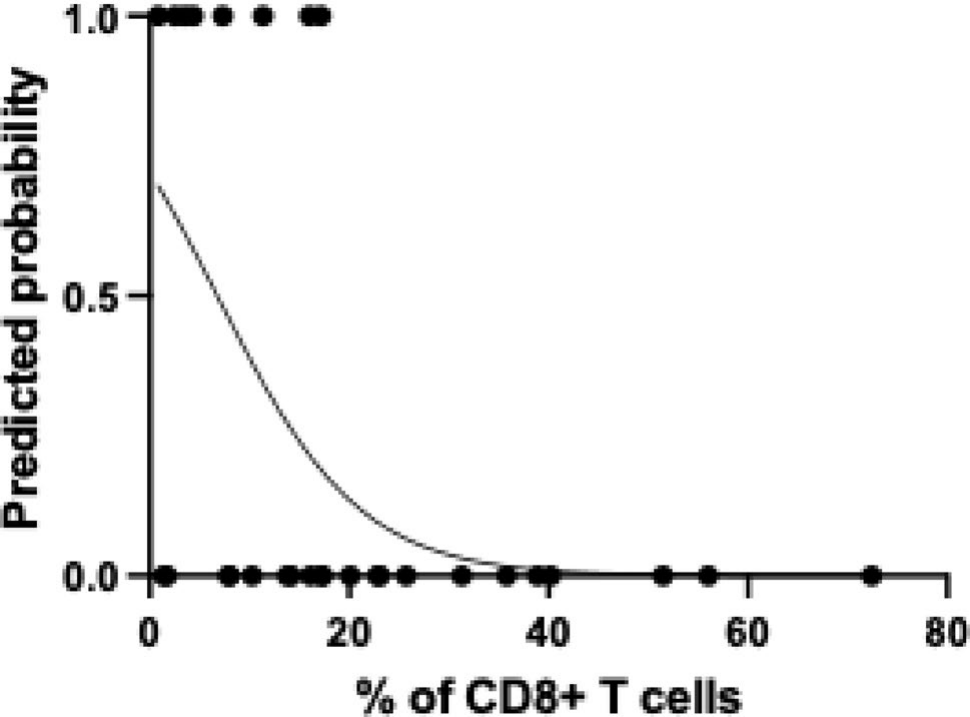
Logistic regression analysis of the association between the percentage of CD8 + T cells and the likelihood of a positive axillary lymph node. The x-axis represents the percentage of CD8 + T cells, and the y-axis shows the predicted probability of a positive axillary lymph node. The solid curve represents the predicted probabilities based on the univariate logistic regression model, which yielded an odds ratio of 0.87 (95% CI: 0.74–0.96)

Considering this strong association, we performed a preliminary analysis to assess the impact of CD8 + T cell percentage on the prediction of axillary metastasis using logistic regression (Fig. 4). This analysis revealed an odds ratio of 0.87 (95% CI: 0.74–0.96), suggesting that the increase in CD8 + T cells is associated with reduced likelihood of a positive axillary lymph node.

### Effect of TIL subtypes on long term survival of TNBC

**Fig. 5:**
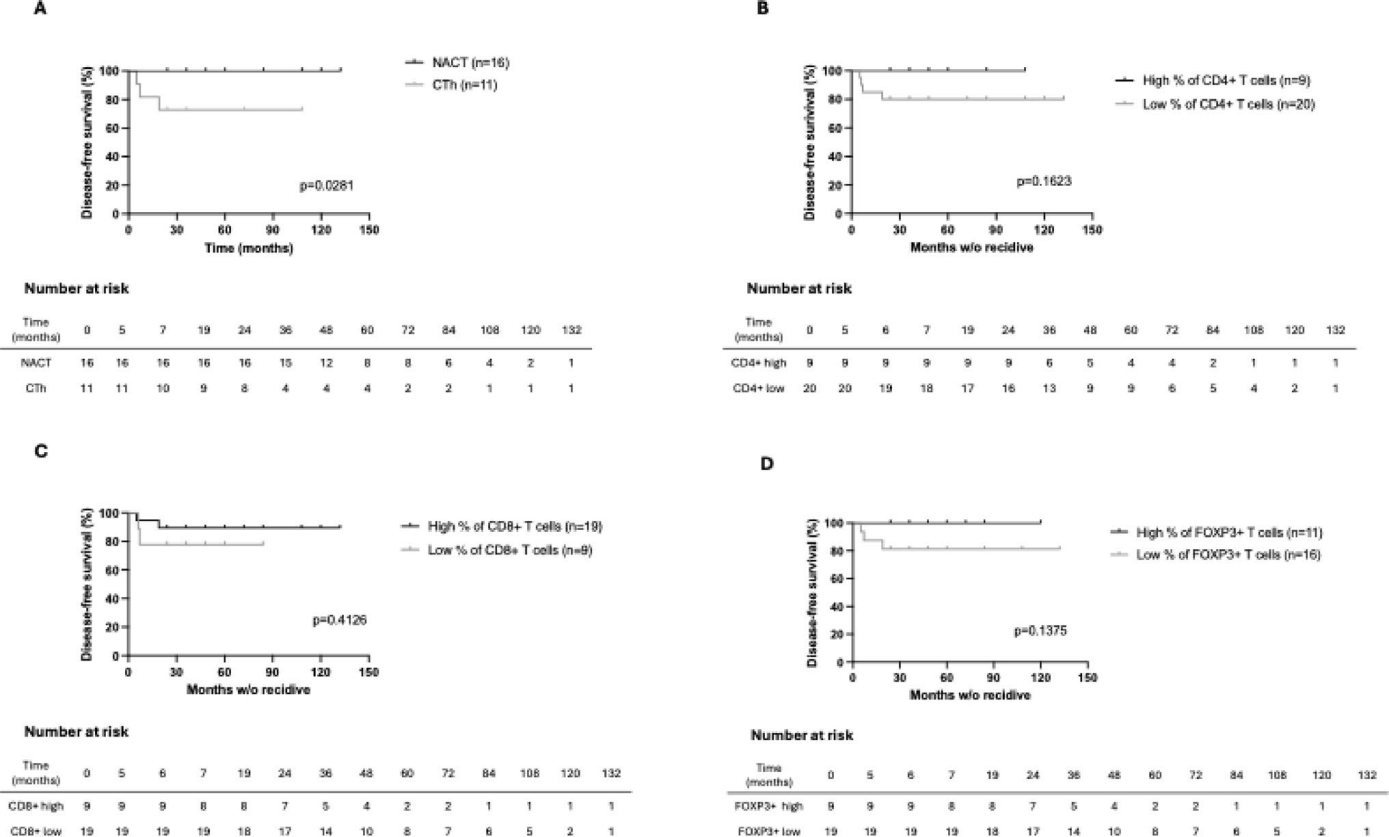
Kaplan-Meier survival curves of disease-free survival. Survival differences were compared across groups using the log-rank test. Panel A: neoadjuvant chemotherapy (NACT) versus chemotherapy (CTh); Panel B: High (≥ 72.6%) versus low (< 72.6%) percentage of CD4 + T cells; Panel C: High (≥ 9.12%) versus low (< 9.12%) percentage of CD8 + T cells; Panel D: High (≥ 3.77%) versus low (< 3.77%) percentage of FOXP3 + T cells

Disease-free survival was used as the primary prognostic endpoint and was estimated using the Kaplan-Meier survival method and, as described before, dividing the cases into groups dichotomized by type of chemotherapy (CTh) (NACT or adjuvant chemotherapy, Fig. 5A) and in low *(< ) or high (≥) levels of TIL* (Fig. 5B, C and D). As shown in Fig. 5A., patients who underwent NACT showed a significant improvement in DFS compared to those who received adjuvant chemotherapy (*p* = 0.0281). *When analyzing DFS according to TIL percentages*,* higher levels of CD4 + T cells were associated with a tendency toward increased DFS*,* although not statistically significant (p* *= 0.1623*, Fig. 5B*). Similarly*,* higher levels of CD8 + T cells and FOXP3 + T cells (*Fig. 5C and D*) showed trends toward improved DFS (p* *= 0.4126 and* *p* *= 0.1375*,* respectively)*,* but these differences did not reach statistical significance.*

## Discussion

The complex interplay between cancer cells and the immune system has been extensively studied in recent years, revealing that the immune system plays a major role in cancer progression and prognosis. Additionally, malignant cells can influence their immune microenvironment, creating conditions that promote tumor growth and metastasis [23]. A positive correlation exists between TIL infiltration and prognosis in TNBC [8, 24], which intensified the efforts to understand the roles of different subpopulations, as they significantly impact on prognosis and treatment response [25].

Among the TIL subsets, CD8 + T cells are particularly associated to improved patient survival in TNBC [26]. The roles of CD4 + T cells and FOXP3 + remain unclear, as studies have linked them to both positive [25] and a potentially negative prognostic outcomes [27].

Regarding NACT, TIL levels serve as markers of an active antitumor immune response and are independent predictors of response to therapy, as well as favorable prognostic factors in TNBC [28]. Neoadjuvant treatment facilitates pathological response-guided adjuvant therapy, which can enhance survival. Therefore, achieving a pCR is confirmed to be the most valuable biomarker for survival outcomes in the neoadjuvant setting. In our study, *we initially observed a tendency of higher levels of CD4 + T cells in individuals achieving pCR. This association was confirmed and strengthened when optimizing the groups according to CD4 + T cells levels*,* revealing that high CD4 + T cell counts were significantly associated with* pCR achievement compared to the no pCR group. Previous studies have reported that higher CD4 + T cells and CD8 + T cells are linked to improved responses to systemic treatment in breast cancer [29]. Consistent with our findings, neoadjuvant chemotherapy resulted in a significantly higher pCR rate associated with CD4 + T lymphocytes [8]. Moreover, high intratumoral and stromal CD4 + T cell density has been shown to be a significant predictor for a pCR, independent of the treatment [30, 31]. *Jamiyan at al. have reported that higher intratumoral CD4⁺ T-cell infiltration is significantly associated with a favorable prognosis* *with significant longer relapse free survival in multivariate analysis* [32].

Our findings indicated no significant pCR associated with high levels of CD8 + and FOXP3 + lymphocyte subgroups. Previous reports suggested that FOXP3 + T cell infiltration predicts worse prognosis by mediating tumor immune escape [27]. A study by Abdelrahman et al. demonstrated that the absence of FOXP3+/Tregs is associated with pCR [33]. In line with previous reports, Miyashita et al. found a higher pCR rate in patients with a high CD8+/FOXP3 + ratio compared to those with a low ratio [34]; however, FOXP3 was not individually linked to pCR rates.

We also conducted a correlation analysis between different immune cell infiltrates, which revealed a positive correlation between CD8 + T cells and CD4 + T cells associated with achieving pCR, a conclusion previously reported [18, 30]. According to our results, positive correlations between FOXP3 + and both CD8 + T cells were linked to pCR achievement. This work underscores the critical role of the immune microenvironment in response to neoadjuvant chemotherapy.

In this study, we evaluated the association between levels of CD4 + T cells, CD8 + T cells and FOXP3 + lymphocyte subpopulations and their association with axillary metastasis. TIL have shown a significant correlation with lymph node status in breast cancer. Specifically, an increased number of TIL is associated with a lower incidence of lymph node metastases in both early-stage and locally advanced breast tumors. In our analysis, patients without positive axillary lymph nodes tended to have higher percentages of CD8 + T cells; however, this association did not reach statistical significance in the initial test (*p* = 0.667). Nonetheless, subsequent analyses, including group comparisons according to axillary lymph node status and logistic regression, demonstrated a significant association between higher CD8 + T cell levels and a lower likelihood of positive axillary lymph nodes, supporting a potential link between TIL levels and lower tumor aggressiveness. Moreover, a recent study indicated that high TIL levels were related to axillary pCR and could help identify appropriate patient groups for less aggressive treatments [35].

 Tomioka et al. reported a better prognosis in DFS associated with the presence of CD8⁺ and FOXP3⁺ T cells in both primary tumors and metastatic lymph nodes [36]. In contrast,* no relationship was found between the number of FOXP3⁺ TIL and axillary lymph node* [37].

The KI67 marker of cell proliferation rate has been explored as a biomarker of aggressive and metastatic TNBC cases with poor outcome [38]. In our study, we found a strong negative correlation (ρ = -0.8469; *p* = 0.0246) between CD8 + T cells and high KI67 in cases without pCR.

On the other hand, a positive strong correlation was identified between the percentage of CD4 + T cells and high KI67 (ρ = 0.8469; *p* = 0.0246).

While these are preliminary results, and considering the observed association between CD8 + T cells and absence of axillary metastasis, these data suggest that in the absence of pCR, high KI67 is associated with lower CD8 + T cells increasing the evidence on the potential of CD8 + T cells to predict tumor aggressiveness and poor prognosis.

According to long-term prognosis, our results indicate that patients with high TIL tend to have improved DFS across all TIL subtypes. Although none of these associations reached statistical significance (CD4 + T cells, *p* = 0.1623; CD8 + T cells, *p* = 0.4126; FOXP3 + T cells, *p* = 0.1375), the observed trends are consistent with previous conclusions [20, 27], indicating that elevated levels of CD4+,* CD8+*,* and FOXP3 + T cells may be associated with better long-term outcomes in patients with TNBC* [39].

The presence of TIL may help in identifying patients eligible for systemic therapy de-escalation, given their association with improved pCR rates, response to chemotherapy, and long-term prognosis [40].

In our study, 16.7% of patients were positive for BRCA 1/2 pathogenic or probably pathogenic variants, with no impact on treatment.

Several limitations were identified, particularly being a single-institution study with a small sample size. However, the data collected in this study provide relevant insights that can support the design of larger, multi-institutional studies, serving as a foundation to further explore the potential of TIL in predicting short- and long-term outcomes in TNBC.

An important methodological limitation of our study is the use of TMAs with single 500 μm ROIs per case, which may not fully capture the spatial heterogeneity of TIL or the complete stromal compartment within the primary tumor. Current recommendations from the International TIL Working Group emphasize scoring stromal TIL on whole tissue sections; due to the retrospective design and the use of pre-existing TMAs, we were not able to systematically perform this assessment for all cases. Our approach, however, is in line with previous TMA-based investigations of TIL in breast cancer and was carefully standardized by selecting ROIs at the invasive front with preserved tumor–stroma interface. Future studies using whole-slide digital quantification of stromal TIL in larger cohorts will be important to validate and extend our findings.

Adoptive cell therapy is currently being investigated in early-phase clinical trials for breast cancer; however, there is no conclusive evidence of its efficacy yet. Several early-phase trials are exploring the TIL therapy in TNBC, HER2-positive and luminal-type metastatic breast cancer (NCT04111510, NCT01462903, NCT01174121, NCT00301730).

In conclusion, high levels of specific TIL phenotypes (CD4 + T cells, CD8 + T cells, and FOXP3+) predict positive short-term and long-term prognosis for TNBC. Our findings indicate that higher CD4⁺ T-cell counts are significantly associated with achieving pCR. TIL represent a promising potential biomarker due to their prevalence in TNBC and their association with improved clinical outcomes, although they have yet to be incorporated into clinical practice. A significant correlation between TIL and lymph node status was also observed, demonstrating a protective effect in the presence of high CD8 + T cells levels in logistic regression analysis. Patients with high TIL infiltration have a lower risk of recurrence and are associated with better pCR rates following neoadjuvant therapy, making them valuable for identifying potential candidates for therapy de-escalation. Furthermore, the role of TIL in immunotherapy is expected to grow in the future, warranting further research.

## Supplementary Information

Below is the link to the electronic supplementary material.

Supplementary Material 1
