# Supplementary material for: Prognostic relevance of specific TIL (CD4+, CD8+, and FOXP3 + T-cell infiltrates) in triple-negative breast cancer: short- and long-term outcomes
Source: Breast Cancer. 2026 Jan 7;33(2):386–95. doi: 10.1007/s12282-025-01819-y (PMC12960379; doi:10.1007/s12282-025-01819-y)
Supplement: Supplementary file 1 — Supplementary Material 1 [file 12282_2025_1819_MOESM1_ESM.docx]

Supplementary data


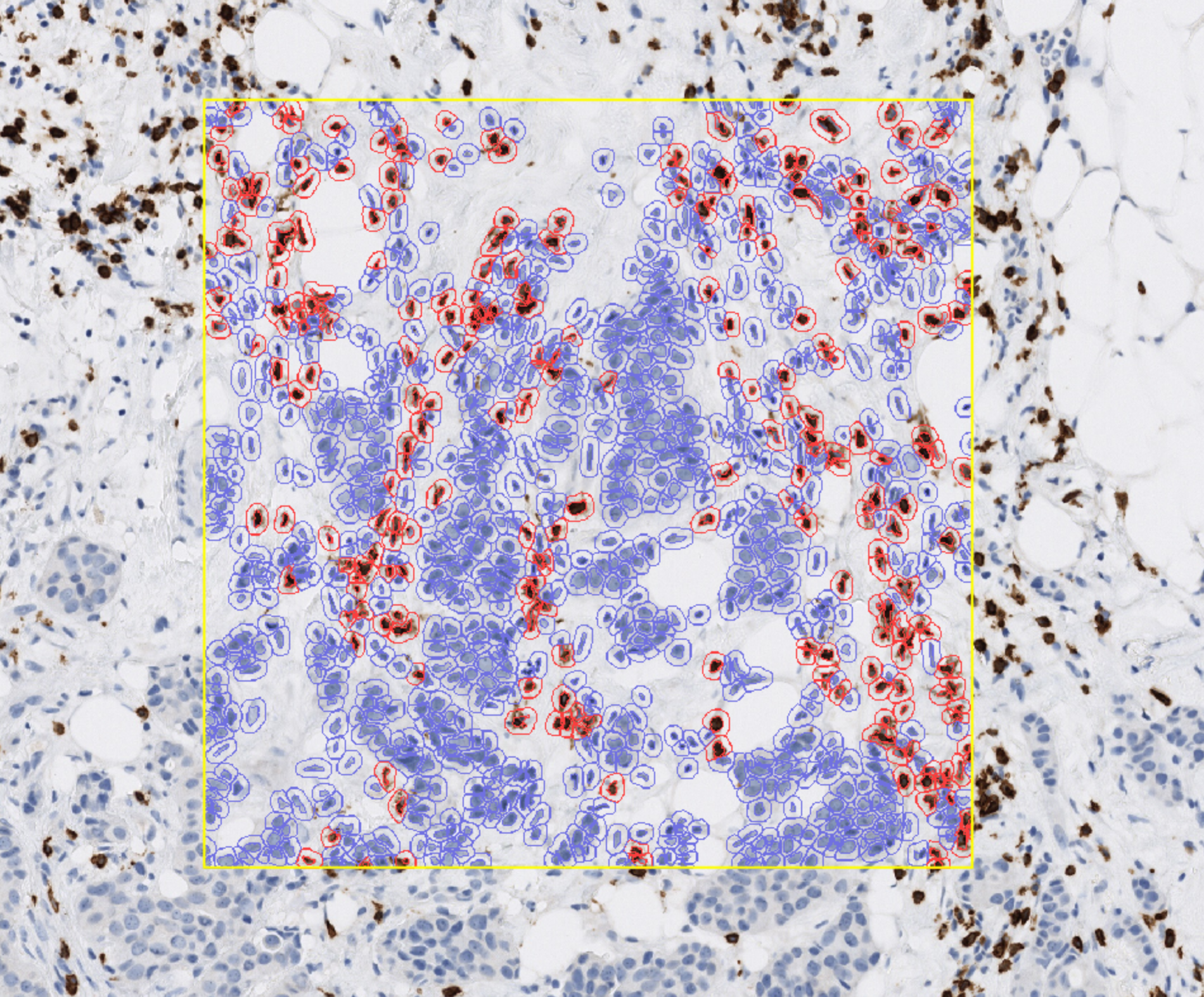


**Figure S1**— Example of digital quantification of CD8-positive lymphocytes.

Representative 500 × 500 μm region of interest (ROI) analyzed using QuPath. Slides were scanned at 20× (0.49 μm/pixel). Positive cells (DAB-stained) are shown in red and negative nuclei in blue after automated nuclear detection and classification

**Table S1. Correlations between tumor-infiltrating lymphocytes (TIL) subtypes and Ki67 expression in triple negative breast cancer (TNBC) patients**. Spearman correlations between the percentage of TIL subtypes and the KI67 expression among themselves, were calculated separately for individuals with pathological complete response (pCR) (n=10) and without (no) pCR (n=7). Statistically significant associations are highlighted in bold.

|  | **Variables** | | **CD4** | **CD8** | **FOXP3** | **KI67** |
| --- | --- | --- | --- | --- | --- | --- |
| **pCR** | **CD4** | ρ |  | **0.7455** | 0.6500 | 0.1534 |
|  |  | *p-value* |  | *0.0174* | *0.0666* | *0.6716* |
|  | **CD8** | ρ |  |  | **0.7833** | -0.0491 |
|  |  | *p-value* |  |  | *0.0172* | *0.8963* |
|  | **FOXP3** | ρ |  |  |  | -0.0760 |
|  |  | *p-value* |  |  |  | *0.8687* |
| **No pCR** | **CD4** | ρ |  | **-0.8929** | 0.0000 | **0.8469** |
|  |  | *p-value* |  | *0.0123* | *1.0000* | *0.0246* |
|  | **CD8** | ρ |  |  | 0.2857 | **-0.8469** |
|  |  | *p-value* |  |  | *0.5560* | *0.0246* |
|  | **FOXP3** | ρ |  |  |  | 0.1441 |
|  |  | *p-value* |  |  |  | *0.7603* |
|  |  |  |  |  |  |  |
